# SPARC is expressed in scars of the Tenon’s capsule and mediates scarring properties of human Tenon’s fibroblasts in vitro

**Published:** 2011-01-19

**Authors:** Rudolf Fuchshofer, Ulrike B. Kottler, Anne V. Ohlmann, Ursula Schlötzer-Schrehardt, Anselm Jünemann, Friedrich E. Kruse, Andreas Ohlmann

**Affiliations:** 1 Institute of Human Anatomy and Embryology, University of Regensburg, Regensburg, Germany; 2 Department of Ophthalmology, University Erlangen-Nürnberg, Erlangen, Germany; 3 Department of Ophthalmology, Ludwig-Maximilians-University, Munich, Germany

## Abstract

**Purpose:**

To investigate the expression of the matricellular protein SPARC (secreted acidic cysteine-rich glycoprotein) in scarred human Tenon’s capsule and in cultured human Tenon’s fibroblasts (HTF), and to analyze the influence of SPARC on cell proliferation and collagen matrix contraction in vitro.

**Methods:**

Human Tenon's capsule scars obtained from surgical revisions after filtration surgery were analyzed for SPARC expression by immunohistochemistry. In cultured HTF cells, SPARC expression was assessed by northern and western blot analyses after incubation with transforming growth factor (TGF)-β1 and TGF-β2. Cell proliferation was determined by bromodeoxyuridine (BrdU)–labeling and HTF cells-mediated collagen matrix contraction by morphometric measurements of three-dimensional collagen lattices after treatment with SPARC and/or TGF-β1.

**Results:**

In scarred human Tenon’s capsule specimens, an increased expression of SPARC was mainly localized to the extracellular matrix and to blood vessel walls as compared to healthy control Tenon’s capsule. In cultured HTF cells, treatment with TGF-β1 more than TGF-β2 induced the expression of SPARC both on the mRNA and protein level. Incubation of HTF cells with SPARC resulted in an increase in collagen matrix contraction and cell proliferation. Moreover, a combined incubation of SPARC and TGF-β1 stimulated HTF cell proliferation significantly over the levels that were observed after single treatment.

**Conclusions:**

Our data provide evidence that SPARC contributes to excessive wound healing and scar formation in human Tenon’s capsules after filtration surgery and may thus represent a novel target for anti-fibrotic strategies.

## Introduction

Glaucoma is one of the leading causes of irreversible blindness worldwide. The estimated number of people bilateral blinded from glaucoma in 2010 will be approximately 8.4 million and 11.2 million in 2020 [1]. Several prospective randomized clinical trials demonstrate that an increase in intraocular pressure (IOP) is the major risk factor for development and progression of glaucomatous optic neuropathy [2]. Therefore filtration surgery is considered to be the most effective method to achieve a sufficient decrease in IOP level when topical treatment or less invasive surgery is ineffective.

The goal of filtration surgery is to establish an artificial outflow for the aqueous humor (AH) via a scleral fistula into the subconjunctival space. The major risk factor for failure of filtrating surgery is excessive post-operative wound healing in the conjunctiva and at the level of the sclerostomy sites such that outflow resistance of the fistula is elevated by increased extracellular matrix (ECM) deposition [3]. To limit the post-operative scarring response, relatively unspecific anti-proliferative agents, such as Mitomycin C or 5-fluorouracil, are used to improve surgical outcomes [4,5]. However, severe side effects have been observed [6]. Therefore analyses of the scarring processes after filtration surgery are ongoing to identify potential alternatives.

The complex wound healing processes following injury of the Tenon’s capsule include proliferation, migration, synthesis of ECM components and collagen contraction by human Tenon’s fibroblast (HTF) cells that are derived from the subconjunctival space. The activation of HTF is mainly mediated by cytokines and growth factors [3,7,8]. After filtration surgery, the wound healing response is thought to be influenced by the passage of AH and its containing growth factors through the surgical wound [9]. The most potent growth factors in the AH to stimulate fibroblast activation are transforming growth factor (TGF)-β1 and TGF-β2 [3,10].

Alongside growth factors and cytokines, matrix modulating matricellular proteins are involved in remodeling processes and turnover of the ECM [11]. Increased expression of these proteins may enhance pathological processes such as scar formation and tissue fibrosis. In the past decade the matricellular protein SPARC (secreted protein acidic and rich in cysteine), also known as osteonectin or BM-40 was shown to be involved in scar formation and in tissue fibrosis [12-20]. In accordance with these observations a lack of SPARC in mice or rats markedly reduces pulmonary [21], renal [22], dermal [23], or hepatic [24] fibrotic processes. But on the other hand in SPARC deficient mice an accelerated wound closure was observed [25]. Nevertheless in a recent study, Seet and colleagues [26] could show that in SPARC knockout mice, the scarring response is diminished after filtration surgery. These reports strongly suggest an important role for SPARC in ECM remodeling during a fibrotic response and scar formation. Moreover, SPARC like other matricellular proteins also has the distinct potential to modify the signaling of growth factors such as that of TGF-β via an increased phosphorylation of Sma and Mad related protein (Smad) 2 [27,28].

Therefore we investigated the expression of SPARC in human Tenon’s capsule scars after filtration surgery and in HTF cells after incubation with TGF-β1 and TGF-β2. We also studied proliferation and collagen matrix contraction in SPARC-treated HFT cells as markers for scar formation in vitro. We found that SPARC is expressed in human Tenon’s scars and is substantially induced in HTF cells after treatment with TGF-β. Furthermore, proliferation and collagen matrix contraction are increased in SPARC-treated HTF cells. Our results provide evidence that SPARC may be involved in excessive wound healing of human Tenon’s capsule after glaucoma surgery.

## Methods

### Sample collection and cell culture

Sample collection was approved by the local ethic committee and was performed after informed consent of patients, following the tenets of the Declaration of Helsinki. Biopsies of healthy human Tenon’s capsule and Tenon’s capsule scars were obtained from patients with retinal detachment or primary open-angle glaucoma, respectively, during pars plana vitrectomy or revision surgery after trabeculectomy. Patients with other ocular or systemic disease, such as inflammatory diseases or diabetes mellitus were excluded from the study.

Cultures of human Tenon’s fibroblast were established as described previously [29]. Briefly, Tenon’s capsule biopsies from five different healthy-controls were dissected, placed in 50 ml tissue-culture flasks in Dulbecco's modified Eagle's medium (DMEM/Ham's F12; Invitrogen, Karlsruhe, Germany) containing 15% (v/v) fetal calf serum and antibiotic-antimycotic solution (10,000 U/ml penicillin, 10,000 μg/ml streptomycin and 25 μg/ml amphotericin B; all from Invitrogen), incubated at 37 °C in a humidified 95% air/5% CO_2_ atmosphere and fed every 3 days. Prior to experiments, HTF were starved 24 h in serum free cell culture medium. For all assays, serum free cell culture medium and 2nd-4th passage cells were used.

### Proliferation assay

Cell proliferation assays of human Tenon’s fibroblast were performed by using bromodeoxyuridine (BrdU)–labeling of dividing cells according to manufacturer’s instructions (Roche, Mannheim, Germany). In brief, cells were seeded into 96-well plates at an initial density of 2×10^3^ cells/well and allowed to attach for 24 h. After another 24 h in serum free medium, the cells were treated with various concentrations of human recombinant SPARC and/or activated TGF-β1 (200 pg/ml; both R&D Systems, Wiesbaden, Germany). After 3 days, cells were fixed and incorporated BrdU was detected by enzyme-linked immunosorbent assay (ELISA) using an ELISA plate reader (Tecan, Crailsheim, Germany) at 450 nm.

### Collagen contraction assay

Three-dimensional collagen lattices were prepared as previously described [30]. Briefly, type I collagen from rat tail (Sigma, Dorset, UK) was dissolved at 2 mg/ml in 0.1% acetic acid to create a stock solution. The collagen matrix was quickly prepared on ice by adding 6 ml of collagen stock solution to 3.6 ml of 0.1% acetic acid, 1.2 ml of 10× concentrated DMEM, and 1.2 ml of sodium bicarbonate solution (11.76 mg/ml) for a final concentration of 1 mg/ml collagen. The pH was adjusted to 7.2–7.4 by adding 0.1 mol/l sodium hydroxide solution. Fibroblasts were then added to achieve a final concentration of 5×10^5^ cells/ml of collagen suspension; 500 μl of this suspension was aliquoted into each well of a 24-well culture plate. After incubation for 15 min at 37 °C with 5% CO_2_ for polymerization, the matrix in each well was overlayed with 500 μl of serum-free medium containing 100 ng/ml SPARC. The gels were gently mechanically released from the wall and bottom of the wells with a sterile spatula. Medium was changed every 3 days. Collagen lattices were scanned at various time points and lattice area was analyzed using the Digivision software (Soft Imaging System, Münster, Germany).

### RNA isolation, cDNA generation, and northern blot analyses

After incubation, cells were washed two times with PBS and harvested from cell culture dishes in Trizol (Invitrogen) according to the manufacturer’s recommendations. The integrity of the total RNA was confirmed by gel electrophoresis. First-strand cDNA synthesis was prepared from total RNA using the Superscript cDNA Synthesis Kit (Invitrogen) according to manufacturer’s instructions.

A human *SPARC* cDNA fragment was amplified by PCR using the primer pairs 5′-TGC CTG ATG AGA CAG AGG TG-3′ and 5′-TAC AGG GTG ACC AGG ACG TT-3′, and cDNA of human Tenon’s fibroblasts as template (product length, 465 bp). PCR was performed in a final volume of 50 µl by initial denaturation at 94 °C for 2 min, followed by 35 cycles of 30 s at 94 °C, 45 s of annealing at 55 °C, and 90 s of extension at 72 °C. After the last cycle, the extension time was 10 min. PCR products were gel-purified by using the QIAEX II Gel Extraction Kit (QIAGEN, Hilden, Germany) and cloned into pCRII-Topo vector (Invitrogen). After linearization of the vector with HindIII, antisense RNA probes for *SPARC* were generated and labeled with DIG-11-UTP using T7-polymerase (Roche, Mannheim, Germany).

For northern blot analysis, 10 µg of total RNA of human Tenon’s fibroblasts was separated on a 1% agarose gel containing 6% formaldehyde and blotted onto a positively charged nylon membrane (Roche). After transfer, the blot was cross-linked using a UV Stratalinker 1800 (Stratagene, La Jolla, CA). Prehybridization was performed for 1 h at 60 °C using the Dig EasyHyb-buffer (Roche). After overnight hybridization at 60 °C, membranes were washed for 5 min with 2× SSC and 0.1% SDS at room temperature and 15 min with 0.2× SSC and 0.1% SDS at 70 °C. For detection of hybridization signals, membranes were blocked for 30 min at room temperature in 1% blocking reagent, 0.1 M maleic acid, and 0.15 M NaCl, pH 7.5, and incubated 30 min in anti-digoxigenin-alkaline phosphatase diluted 1:10,000 (Roche). After washing membranes two times for 15 min in 0.1 M maleic acid, 0.15 M NaCl, pH 7.5, and 0.3% Tween-20, chemiluminescence detection was performed (CDP-Star; Roche). The membranes were exposed using a BAS 3000 Imager work station (Fujifilm, Düsseldorf, Germany). To monitor the integrity of RNA, the relative amounts of RNA loaded on the gel and the efficiency of transfer, membranes were stained with methylene blue. For densitometry AIDA Biopackage software (Raytest, Straubenhardt, Germany) was used.

### Protein preparation and western blot analyses

For western blot analyses to detect SPARC, confluent human Tenon’s fibroblasts were starved overnight in serum free medium, and incubated with activated TGF-β1 or activated TGF-β2 (1 ng/ml; R&D Systems, Wiesbaden, Germany) for 3 days. The total cellular protein fraction was dissolved in radioimmunoprecipitation assay (RIPA) buffer and, after homogenization, insoluble constituents were removed by centrifugation. Protein content was measured by bradford colorimetric assay according to manufactures instructions (Thermo Scientific, Germany), and up to 25 µg of cytosolic proteins were subjected to a 10% SDS–PAGE. Separated proteins were transferred on a PVDF membrane (Roche) by semidry blotting. After blocking with 5% low fat milk in PBS-T, the membranes were incubated overnight with mouse-anti-SPARC antibodies (Santa Cruz), diluted 1:1.000 in PBS-T. After washing three times with PBS-T, membranes were incubated with HRP-conjugated goat-anti-mouse secondary antibodies at a 1:10,000 dilution in PBS-T (Millipore, Schwalbach, Germany). Antibody labeling was visualized using the Immobilon HRP substrate (Millipore), documented on a LAS 3000 Imager work station (Fujifilm, Düsseldorf, Germany) and quantified by using the AIDA Biopackage software (Raytest, Straubenhardt, Germany). As loading control and for quantification, a coomassie blue staining was used.

### Immunohistochemistry

For immunohistochemistry, samples of three healthy-control and three scarred human Tenon’s capsules were fixed in 4% PFA for 4 h. After washing with 1x PBS overnight, samples were embedded in O.C.T compound (Sakura Finetek, Zoeterwoude, Netherlands) to process frozen sections according to standard protocols. Before overnight incubation at 4 °C with mouse monoclonal anti-β-SPARC antibodies (1:100; Santa Cruz, Santa Cruz, CA), samples were incubated in 3% BSA (BSA) solution for 30 min. As negative control, sections were incubated with 3% BSA in PBS overnight. After three washes (10 min each) with PBS, samples were treated for 1 h with Alexa 488 Fluor-labeled goat-anti-mouse antibodies (1:1,000; Invitrogen). After three washes, sections were incubated in a 1:10,000 propidium iodide solution (stock 0.33 mg/ml in PBS) for 15 min, washed three additional times and mounted in fluorescent mounting medium. Samples were analyzed on an Axiovision fluorescence microscope (Carl Zeiss, Jena, Germany).

### Statistics

All results are expressed as mean±SEM. Comparisons between the mean variables of 2 groups were made by a 2-tailed Student’s *t*-test. P values less than 0.05 were considered to be statistically significant.

## Results

### SPARC expression is increased in human Tenon’s capsule scars

To analyze if SPARC is expressed in scars of the Tenon’s capsule after filtration surgery, immunohistochemical staining using specific antibodies against SPARC in healthy and scarred human Tenon’s capsule was performed.

**Figure 1:**
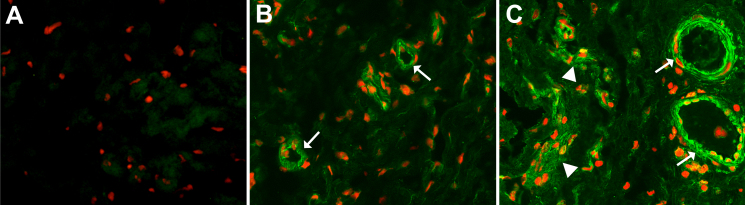
SPARC expression is increased in human Tenon’s capsule scars. Immunohistochemistry for SPARC (green) in scarred human Tenon’s capsule (**C**) shows a marked accumulation of SPARC around and within vessel walls (arrows) and in areas of condensed extracellular matrix (arrow heads), whereas only weak signals are detected in extracellular matrix and vessel walls (arrows) of healthy tissue (**B**). **A**: control immunostaining of a healthy Tenon’s capsule. red: propidium iodide staining; original magnification: 20×.

In the normal human Tenon’s capsule, a weak but specific signal for SPARC was only detected around and within blood vessel walls (Figure 1B). Similar to this observation, in the scarred human Tenon’s capsule, SPARC was also localized around and within blood vessel walls, but the staining intensity was markedly increased when compared to healthy specimens (Figure 1C). Additionally, the blood vessels showed a vasodilation in the scarred human Tenon’s capsule, which is typical for scar tissues. In the normal human Tenon’s capsule, only a weak signal for SPARC was detected within the connective tissue, whereas in scars of the Tenon’s capsule a specific staining was observed throughout the collagenous stroma (Figure 1). In the extracellular matrix of human Tenon’s scars, a prominent localization of SPARC was seen in areas of a denser connective tissue (Figure 1C, arrow heads).

### TGF-β induces the expression of SPARC in human Tenon’s fibroblasts

After filtration surgery, increased levels of TGF-β are a major risk factor for the formation of Tenon’s capsule scars. Therefore we wondered if TGF-β1 and/or TGF-β2 can induce the expression of SPARC in cultured HTF cells.

**Figure 2:**
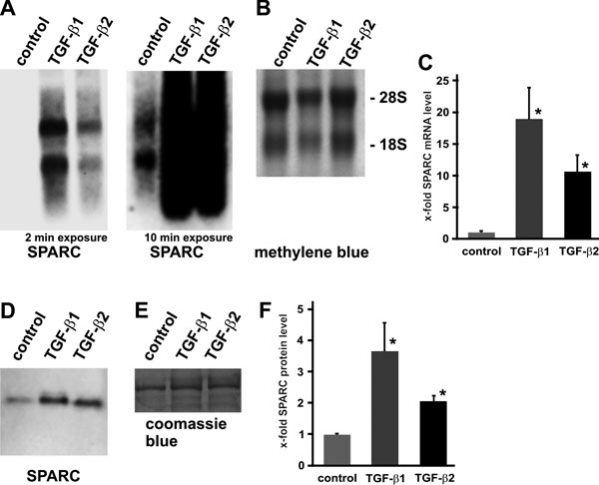
TGF-β induces expression of SPARC in human Tenon’s fibroblasts in vitro. Representative northern blot analysis (**A**, **B**) and densitometry (**C**) for SPARC expression in HTF cells without and with incubation with activated TGF-β1 (1 ng/ml) or TGF-β2 (1 ng/ml) for three days. For quantification, *SPARC* mRNA levels were measured densitometrically, normalized to 28S methylene blue staining and expressed as ×-fold to levels of untreated control cells (mean±SEM of 3 independent experiments; *p<0.05). Representative western blot analysis (**D**, **E**) and densitometry (**F**) in HTF cells for SPARC without and with incubation with activated TGF-β1 (1 ng/ml) or TGF-β2 (1 ng/ml) for three days. For quantification, SPARC protein levels were measured densitometrically, normalized to coomassie blue staining and expressed as ×-fold to levels of untreated control cells (mean±SEM of 3 independent experiments; *p<0.05).

Untreated HTF cells showed only weak expression for *SPARC* mRNA. After incubation of the cells with activated TGF-β1 (1 ng/ml) and -β2 (1 ng/ml) for three days, a marked increased mRNA expression of *SPARC* was observed by northern blot analyses when compared with untreated control cells. In addition, the treatment of HTF cells with TGF-β1 caused an 18.9 fold induction of *SPARC* mRNA and was more potent as that with TGF-β2 that mediates a 10.6 fold increase (Figure 2A-C).

To investigate whether the increase in transcription of *SPARC* mRNA also results in elevated protein level, western blot analyses were performed. In untreated control cells, a basal protein level of SPARC expression was detected, whereas the TGF-β1 (1 ng/ml) and TGF-β2 (1 ng/ml) treatments lead to a higher synthesis rate in HTF cells. Similar to northern blot analyses, we could also observe higher protein levels after treatment of HTF cells with TGF-β1 (3.6 fold) as that after incubation with TGF-β2 (2.1 fold), although it was not such prominent like on the mRNA level. Again, TGF-β1 was more potent than TGF-β2 to induce SPARC protein synthesis (Figure 2D-F).

### SPARC induces proliferation of HTF cells

During wound healing fibroblast cell proliferation is commonly involved in the process of scar formation. To investigate if SPARC can promote these processes, HTF cells were incubated with different concentrations of SPARC and cell proliferation was analyzed by BrdU-ELISA assays.

**Figure 3:**
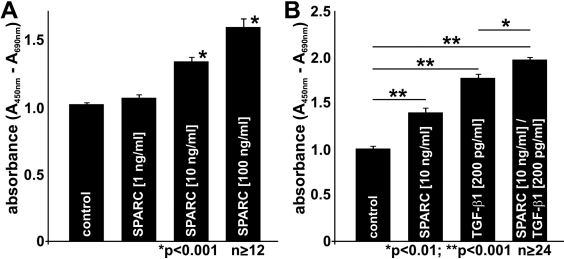
SPARC induces proliferation of HTF cells. For quantification of cell proliferation, HTF were incubated with indicated concentrations of SPARC (**A**) and/or 200 pg/ml TGF-β1 (**B**) in BrdU containing (10 µM) serum free cell culture medium. After three days, incorporated BrdU level were measured by ELISA technique and expressed as relative absorbance to untreated controls (mean±SEM of 2 independent experiments; n≥24; *p<0.01; **p<0.001).

After an incubation of HTF cells with 1 ng/ml SPARC for three days, only a slight increase of cell proliferation was detected, whereas higher concentrations of SPARC significantly induced HTF cell proliferation in a dose dependent manner up to 1.6 fold when compared to untreated controls (Figure 3A).

Because TGF-β1 is a very potent inducer of HTF cell proliferation and also of SPARC expression, we analyzed whether SPARC-mediated HTF cell proliferation is a downstream effect of TGF-β1. Since SPARC can activate the TGF-β signaling cascade [27] and HTF cells express TGF-β1 [31], only moderate concentrations of both factors were used to avoid an overstimulation.

After an incubation of HTF cells with 200 pg/ml activated TGF-β1 for 3 days a statistically significant increase of cell proliferation of about 1.7 fold was observed in comparison to untreated controls. In addition, the proliferation rate of TGF-β1-treated cells was about 40% higher than after treatment with SPARC. When HTF cells were incubated with a combination of SPARC and TGF-β1, cell proliferation further increased about 2.0 fold in comparison to untreated cells and proliferation rate was enhanced by 30% than after treatment with TGF-β1 only (Figure 3B).Therefore our data strongly suggests additive effects of both growth factors.

### SPARC promotes HTF cell-mediated collagen gel contraction

**Figure 4:**
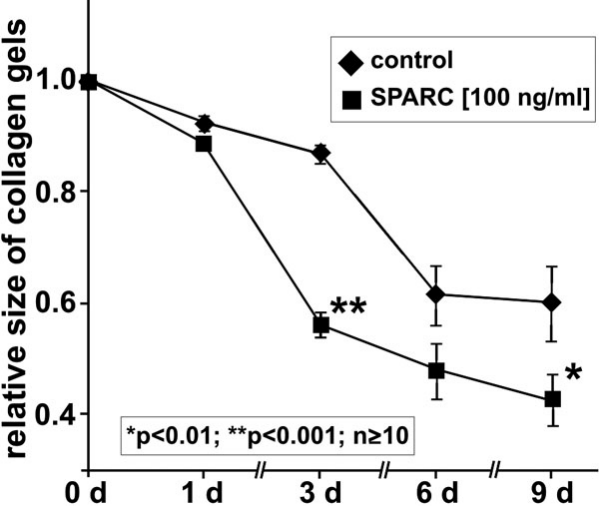
SPARC promotes HTF cell-mediated collagen gel contraction. Effects of 100 ng/ml SPARC on HTF-mediated collagen contraction at different time points from 1 to 9 days. The area of collagen lattices was quantified and plotted as relative size of initial area. Shown are the means±SEM of 2 independent experiments (*p<0.01; **p<0.001; n≥10).

Since contraction of the extracellular matrix is a characteristic step in scar formation, we investigated if SPARC can stimulate this effect in HTF cells on free-floating three-dimensional collagen matrices in vitro. For this purpose HTF cells were incubated with 100 ng/ml SPARC a concentration that was found to induce highest cell proliferation. After day 1 of incubation, SPARC-treated HTF cells as well as untreated controls showed only a slight effect on gel contraction. After continued incubation with SPARC, collagen matrix contraction was markedly enhanced when compared with untreated controls, an effect that was statistically significant after 3 days as well as after 9 days of incubation (Figure 4). In contrast, only a moderate reduction in relative collagen lattice size was observed in untreated control cells over time. These data strongly indicate a substantial contractile effect of SPARC on HTF cells.

## Discussion

In the present study, we demonstrated that SPARC is present in healthy human Tenon’s capsule, where it was restricted to blood vessels, in agreement with findings in other tissues [32]. In scarred human Tenon’s capsules a more intense SPARC staining was observed in blood vessel walls and especially throughout the connective tissue. The higher concentration of SPARC within this tissue could lead to enhanced matricellular effect of SPARC within the Tenon’s capsules.

These findings indicate that SPARC is highly expressed in scarred human Tenon’s capsule and that Tenon’s capsule fibroblasts could be the source of increased SPARC synthesis.

Activation of HTF cells by cytokines and growth factors is considered to be the key effect during the subconjunctival wound healing response [33]. We found that SPARC expression is significantly increased in HTF cells after treatment with TGF-β1 and TGF-β2. Despite the fact that both TGF-β isoforms provoke a higher amount of SPARC as compared to untreated basal levels, we found that TGF-β1 lead to a higher increase of SPARC synthesis compared to TGF-β2. In addition, TGF-β-mediated induction of *SPARC* mRNA was much more pronounced than that of intracellular SPARC proteins. This alleged discrepancy could be explained by different half-lives of *SPARC* mRNA and protein, and by the circumstance that SPARC is secreted and associated with the extracellular matrix.

In the anterior chamber and especially in the AH of normal eyes, TGF-β2 is the predominant TGF-β isoform. Both TGF-β1 and TGF-β2 isoforms are elevated in different types of glaucoma. The concentration of latent/active TGF-β1 is increased in the AH of patients with pseudoexfoliation glaucoma (PEXG) [34], whereas latent/active TGF-β2 is elevated in the AH of patients with primary open-angle glaucoma (POAG). In POAG patients failure of the filtering bleb after surgery is correlated with increased preoperative TGF-β2 levels in AH [35]. Further, Nguyen et al. [36] demonstrated that there is a substantial risk of a breakdown of blood-aqueous barrier after trabeculectomy, which could also contribute to an additional increase of the TGF-β concentration within the AH, especially of TGF-β1, the dominant isoform in the blood. Since TGF-β1 and TGF-β2 are potent factors contributing to an activation of fibroblasts throughout the body, increased amounts of TGF-β1 and TGF-β2 in the AH could be major risk factors leading to a failure of glaucoma filtration surgery by activating HTF cells. The observed increase of SPARC expression within the scarring tissue of the Tenon’s capsule could be mediated by TGF-β.

The activating capability of TGF-β1 and TGF-β2 on HTF cell functions was previously demonstrated by numerous studies. The TGF-β induced activation of HTF cells resulted in an enhanced proliferation rate, an increased migration rate and stronger collagen gel contraction. Our findings showed that HTF proliferation can be also stimulated by SPARC, although its effect was lower than that of TGF-β. However a combined treatment with SPARC and TGF-β1 showed an additive effect suggesting that SPARC contributes to the activation of HTF cells. Since basal and TGF-β-mediated expression of extracellular matrix components is increased in HTF cells of patients having PEX glaucoma or POAG [29], it is of particular interested if this is also true for SPARC expression and hereby a TGF-β independent scarring response could be mediated.

Although the molecular mechanisms of SPARC signaling are still unknown, different studies showed that SPARC treatment provokes activation of latent TGF-β [27,28]. Consistently, an enhanced phosphorylation of Smad2 was observed after SPARC treatment [37], showing that the increase in active TGF-β also led to an enhanced TGF-β signaling. Therefore the additive effect of SPARC on TGF-β-mediated cell proliferation might be also partially due to an enhanced activation of TGF-β.

In addition to increased proliferation, activated fibroblasts mediate contraction of connective tissue constituting another critical step in scar tissue formation. An assay to test whether a substance is contributing to this aspect of the wound healing process is a collagen gel contraction assay. We showed that SPARC can stimulate contraction of collagen gels by HTF cells and thereby contribute to the scarring process.

SPARC is known to be a collagen-binding matricellular protein and its expression is found to be elevated during wound healing processes [38] and in tissues with fibrotic disorders [12,20,39-43]. The high affinity binding site of SPARC for collagen type I, type III, and type IV is localized in the third domain of the SPARC protein and contains two high affinity Ca^2+^-binding EF hands [44]. The interaction between SPARC and collagen suggests that SPARC plays an important role during ECM assembly. This assumption was supported by the finding that the skin of SPARC^−/−^ mice has approximately half the amount of collagen as compared to wild type littermates [45]. A decrease in collagen was also apparent in the heart and in fat deposits of SPARC^−/−^ mice [46,47]. Therefore, an increased amount of SPARC within Tenon capsule together with higher amounts of TGF-β, which is known to increase matrix production in HTF cells, could amplify the scarring process in the Tenon’s capsule following filtration surgery. In accordance with this, in the SPARC^−/−^ mice, a pivotal role of SPARC in the organization of the extracellular matrix in the surgically induced fistula was demonstrated. In these mice a reduced post-operative subconjunctival scarring reaction was observed [26].

In summary, our data reveals for the first time that SPARC is highly expressed in scarred human Tenon’s capsules in vivo and that SPARC can directly induce and amplify the effect of TGF-β on HTF cell proliferation and collagen gel contraction in vitro. Together with observations in SPARC^−/−^ mice after filtration surgery it is tempting to speculate that a modulation of SPARC expression could be a feasible therapeutic approach to reduce excessive scarring and subsequently to prevent failure of glaucoma filtration surgery.
